# A Non-Intrusive Cyber Physical Social Sensing Solution to People Behavior Tracking: Mechanism, Prototype, and Field Experiments

**DOI:** 10.3390/s17010143

**Published:** 2017-01-13

**Authors:** Yunjian Jia, Zhenyu Zhou, Fei Chen, Peng Duan, Zhen Guo, Shahid Mumtaz

**Affiliations:** 1 College of Communication Engineering, Chongqing University, Chongqing 400044, China; chenfei@cqu.edu.cn (F.C.); duanpeng@cqu.edu.cn (P.D.); 2 School of Electrical and Electronic Engineering, North China Electric Power University, Beijing 102206, China; 3 Guoxin Tendering Group Co., Ltd., Beijing 100044, China; guozhen@chinabidding.com.cn; 4 Instituto de Telecomunicações, Campus Universitário de Santiago, Aveiro 3810-193, Portugal; smumtaz@av.it.pt

**Keywords:** passive cyber physical social sensing (CPSS), wireless fidelity (Wi-Fi), people behavior tracking, non-intrusive

## Abstract

Tracking people’s behaviors is a main category of cyber physical social sensing (CPSS)-related people-centric applications. Most tracking methods utilize camera networks or sensors built into mobile devices such as global positioning system (GPS) and Bluetooth. In this article, we propose a non-intrusive wireless fidelity (Wi-Fi)-based tracking method. To show the feasibility, we target tracking people’s access behaviors in Wi-Fi networks, which has drawn a lot of interest from the academy and industry recently. Existing methods used for acquiring access traces either provide very limited visibility into media access control (MAC)-level transmission dynamics or sometimes are inflexible and costly. In this article, we present a passive CPSS system operating in a non-intrusive, flexible, and simplified manner to overcome above limitations. We have implemented the prototype on the off-the-shelf personal computer, and performed real-world deployment experiments. The experimental results show that the method is feasible, and people’s access behaviors can be correctly tracked within a one-second delay.

## 1. Introduction

Cyber-physical systems (CPSs) have emerged as a promising research paradigm [1] which integrates computing, communication and control, that has become a new generation intelligent system. With the extensive development of network applications, CPS has been further integrated, which facilitates the seamless integration between networks and human society. In this context, people, machines, and information systems need to be urgently integrated [2,3,4], which leads to the creation of cyber-physical-social systems [5,6]. On the other hand, with the extensive penetration and integration of personalized mobile devices (e.g., wearable devices, smartphones) in people’s daily lives, people have become the most sensitive social sensors. Therefore, sensing people’s social information has become a new paradigm of cyber physical social system-related applications, called cyber physical social sensing (CPSS) [7,8]. 

CPSS is designed to operate in conjunction with and in service of people. It leverages the sensed information collected by sensing nodes and aggregates it for recognizing people’s behaviors (e.g., mobility pattern), and in turn provides people with a higher level of combined information or services, which simplifies people’s lives. The rapid development of mobile devices with various sensors is the catalyst to promote CPSS to form a context-aware or social-aware mobile wireless sensor network. Many open issues have been studied, such as energy efficiency optimization [9], security attacks [10,11], etc. Among them, the technology utilized to sense or track people’s behavior attracts more attention. CPSS allows people to collect and share information using cyber devices intuitively [12]. In contrast to it, in this article, we explore how to track people’s behaviors without people actively participating in the activity, namely, passive tracking.

Comparisons between different people tracking technologies are summarized in Table 1. Due to many attractive features, such as non-intrusive (third-party) and high positioning precision, camera networks are widely used for tracking people’s behaviors [13,14]. However, camera-based methods require line of sight (LOS), and cannot be deployed flexibly. Radio frequency identification (RFID) [15] and IEEE 802.15.4/ZigBee sensor networks [16] can also be utilized as tracking methods. The tracking systems based on them use electronic tags carried by people, and tag readers deployed in the area of interest. They can perform in non-line of sight (NLOS) manner and can be deployed flexibly. However, all these technologies are intrusive.

Given the popularity of mobile devices today, there has been a growing interest in tracking people’s behaviors through all kinds of sensors built into mobile devices [17], such as global positioning system (GPS), Wireless Fidelity (Wi-Fi), and Bluetooth, etc. GPS has been used in people tracking [17] in a participatory manner, however, it rarely works indoors. Bluetooth-based tracking methods [18] are available indoors, and the tracking mechanism is similar to that of RFID or ZigBee, therefore, the method is also considered intrusive. In addition, cellular signals can be also used to track people’s behaviors [19,20], and the tracking is performed on the network side. However, there exist many limits in this method, such as intrusiveness, poor positioning precision, and inflexible deployment. Wi-Fi has been ubiquitously deployed, especially in public areas such as airports, shopping centers, etc. In addition, almost all of the mobile devices integrate Wi-Fi functionality. In this context, Wi-Fi-based sensing networks have become the world’s largest wireless sensor networks. Researchers have also utilized Wi-Fi to do participatory CPSS [21]. However, in our work, we focus on passive CPSS using Wi-Fi.

Wi-Fi-enabled mobile devices, carried by people, discontinuously send out Wi-Fi messages, even when not connecting with any access point (AP). The presence of people can be identified by sensing these messages. Furthermore, each Wi-Fi-enabled mobile device is equipped with a wireless network adapter, which contains a universal unique device identifier, a media access control (MAC) address. Almost all of the Wi-Fi messages encapsulate the MAC address, with which different people can be distinguished [22]. By placing a dedicated set of hardware devices called monitors dispersed in areas of interest to sense Wi-Fi messages, people’s behaviors can be tracked. One typical application is Wi-Fi-based indoor localization [23], in which Wi-Fi received signal strength (RSS) is utilized by Wi-Fi monitors to identify the locations of people with Wi-Fi devices.

To show the feasibility of the Wi-Fi-based passive CPSS approach, we performed a case study that tracked people’s access behaviors in Wi-Fi networks. Analyzing people’s access behaviors has attracted significant attention recently [24,25,26,27,28,29,30,31,32,33,34,35]. This can be beneficial in many aspects, such as assessment of wireless network utilization, site planning, and design for intelligent and robust wireless network protocols. Therefore, there is a pressing need to characterize and understand people’s access behaviors, including access or exit time, session duration, and other access details, etc.

Previous studies have focused on mobility and association pattern analysis based on the traces collected from Wi-Fi networks through different methods. These methods can be mainly classified into four categories: wired monitoring, polling based on simple network-management protocol (SNMP), specialized applications on the Wi-Fi device, and AP syslog. Wired monitoring collects Wi-Fi traffic at the wired portion adjacent to APs. Schwab et al. [24] presented a method to capture and analyze traffic patterns on a campus Wi-Fi network, based on the traces collected via wired monitoring. Wired monitoring can provide accurate and detailed traces. However, the monitor has to build physical connections with the networks, making this approach inconvenient to deploy. In addition, wired monitoring provides very limited visibility into MAC-level transmission dynamics. The approach we propose adopts passive monitoring, which means the monitors can be deployed flexibly without having to build any connection with the network. SNMP polling is another popular method. Based on the SNMP traces, user mobility patterns in a large corporate Wi-Fi networks were explored in [27]. The study in [30] examined the utilization of Wi-Fi hotspot networks based on the SNMP traces. However, SNMP polls data typically at intervals of minutes. Therefore, some instantaneous transmission dynamics might be lost. Wi-Fi devices with specialized applications can also participate in the trace collection [32]. However, this method suffers from some limitations. First, the assumption that people are willing to install these applications is not always true. Second, these applications may involve modifying drivers, which is complex and costly. Furthermore, the applications run in the Wi-Fi device may need sufficient storage capacity, and incur high energy consumption. Researchers have also used AP syslog [33,35] to characterize and analyze access patterns. However, it is insufficient to expose the MAC-level transmission dynamics of Wi-Fi networks, such as the retransmission and some other details of transmission events.

Some works utilized these methods simultaneously. Researchers in [25] analyzed user behavior and network performance in a public-area wireless network based on traces from SNMP and wired monitoring. In [26] researchers analyzed the usage of a mature campus Wi-Fi network based on traces from syslog, SNMP polling, and tcpdump sniffers. Furthermore, some researchers [28,29,31] have modeled user mobility and association patterns in university campus Wi-Fi networks, based on the traces from SNMP and AP syslog. However, none of them can sense the transmissions from Wi-Fi devices which do not build connection with any AP.

In this article, we develop a passive CPSS system operating in a non-intrusive, flexible, and simplified manner to tracking people’s access behaviors in Wi-Fi networks. Through this system we ease the way to collect detailed access traces with low cost and high flexibility such that brings us closer to understanding people’s access patterns and behaviors in Wi-Fi networks. The contributions are summarized as follows:

- We propose a Wi-Fi based passive CPSS approach for tracking people’s behaviors. The approach works in a non-intrusive, flexible, and simplified manner, and has the ability to provide deep visibility into MAC-level transmission dynamics between Wi-Fi devices and APs. Specifically, it tracks people’s behaviors by sensing Wi-Fi messages from mobile devices people carry with. By extracting the information from these Wi-Fi messages, more data can be obtained.
- Based on the approach, we design a non-intrusive CPSS system. The system works as a third-party monitor, and can be deployed conveniently and cost-effectively. To show the feasibility, we use the system to track people’s access behaviors in Wi-Fi networks. We design the system architecture, and propose a two-sized sliding window algorithm, with which the unreliable information, caused by the loss and retransmission of the 802.11 frame, can be eliminated from the tracked traces. In addition, we design a policy to judge the validness of an access operation.
- We implement the system on the off-the-shelf PC, without changing the hardware or firmware. We also evaluate the system in real Wi-Fi networks. The results show that the system can track people’s access behaviors accurately.

The rest of this article is organized as follows: Section 2 introduces the system description and some definitions. The architecture and implementation details of the proposed system are given in Section 3. In Section 4, the prototype of the system and the experimental results are presented. Finally, we present our conclusions and outlook in Section 5.

## 2. System Description

**Figure 1:**
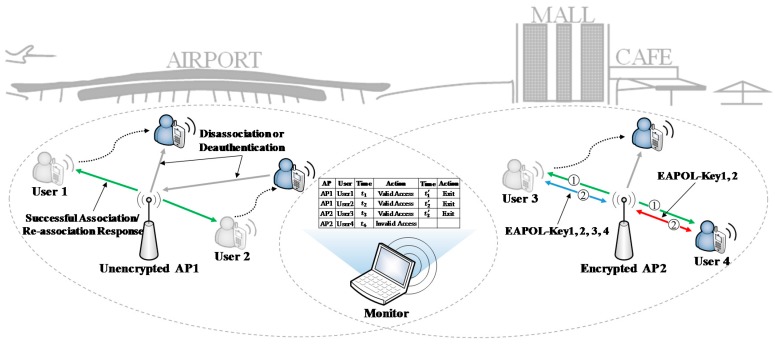
Description of formulating people’s access behaviors in Wi-Fi networks.

The target Wi-Fi networks we consider in this article are unencrypted or encrypted by Wi-Fi protected access or Wi-Fi protected access 2 in pre-shared key mode (WPA/WPA2-PSK), which are ubiquitously being deployed in airports, shopping malls, and cafes, etc. As shown in Figure 1, the system we designed works as a third-party monitor. It tracks the access behaviors of nearby Wi-Fi users, and extracts the access information, including access time, exit time, and some other details. The extracted information will be elaborated in detail subsequently, as illustrated in Section 3.3.

**Figure 2:**
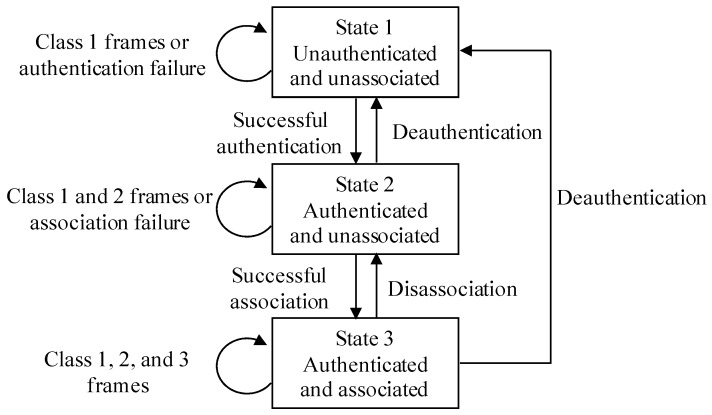
Overall 802.11 state diagram.

For the convenience of description, we give the common 802.11 state diagram first, as shown in Figure 2. A Wi-Fi device usually works in three states, and each state is a successively higher point in the development of an IEEE 802.11 connection. Frames are also divided into different classes. The Wi-Fi device starts in state 1, and data can be transmitted through a distribution system only in state 3. The Wi-Fi device changes the state by exchanging different frames with the AP. When a device tries to access an AP, it exchanges the Authentication frame with the AP first, and then sends the Association/Re-association Request frame to the AP, the AP replies with the Association/Re-association Response frame. The Disassociation or De-authentication frame can be used to abort the connection.

We make some definitions about people’s access and exit operations from an AP, as illustrated subsequently. For the sake of simplicity, we define Association/Re-association Response frame and Disassociation/De-authentication frame as the access frame and exit frame, respectively. We define the access frame and the exit frame reception time on the monitor side as the approximation of the actual occurrence time of the access and exit event on the Wi-Fi device side. Therefore, there is a delay between them. The delay changes under different wireless environment. For example, the delay may get longer if the wireless networks congest, or vice versa.

**Figure 3:**
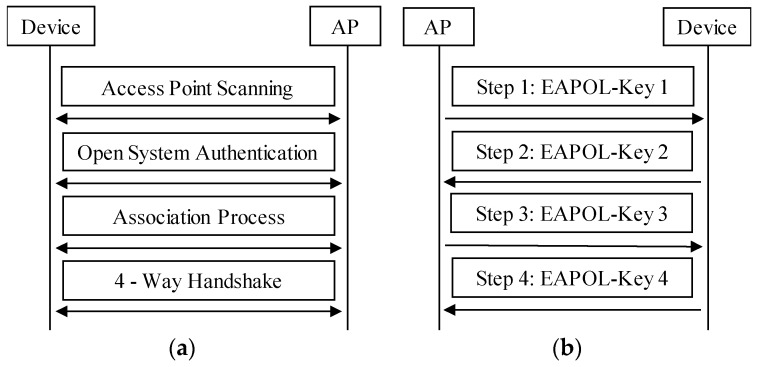
Procedures of accessing an encrypted AP. (**a**) Access procedures; (**b**) The 4-way handshake.

Figure 3a shows the procedures of accessing an encrypted AP. The Wi-Fi device scans the AP first. After finding the AP, the device tries to build a connection with the AP, and it starts with authentication with the AP. Open system authentication can always succeed. Then, the device sends association/re-association request to the AP, and the AP replies with the access frame. Whether an association operation succeeds or not is denoted by the Status Code field in the header of the access frame. The Status Code field is set to 0 when an association/re-association operation succeeds and nonzero on failure. If the association/re-association succeeds, there is a process of 4-way handshake between the AP and the device, which is used for key exchange. The device can exchange data with the AP only if 4-way handshake succeeds.

Figure 3b illustrates the process of 4-way handshake. The AP distributes keys to the Wi-Fi device using extensible authentication protocol over LAN-key (EAPOL-Key) messages encapsulated in quality of service (QoS) Data frames. If 4-way handshake fails, there are several execution loops including steps 1 and 2 in Figure 3b, without step 3 and 4. Then the device or the AP sends the exit frame to the other side to abort the connection. Note that the access procedures for a Wi-Fi device in unencrypted Wi-Fi networks only include access point scanning, open system authentication, and association/re-association process in Figure 3a.

However, the access operation for a Wi-Fi device is not always valid. When a device tries to access an encrypted AP, if the 4-way handshake fails, the device still cannot exchange data with the AP, although the association/re-association succeeds. Therefore, the access operation for the device is invalid. If a Wi-Fi device associates with an unencrypted AP successfully, the access operation is valid. If a Wi-Fi device associates with an encrypted AP successfully, and the 4-way handshake succeeds, then the access operation for the device is valid. However, if the 4-way handshake fails, though the association succeeds, the access operation for the device is invalid.

## 3. System Design

**Figure 4:**
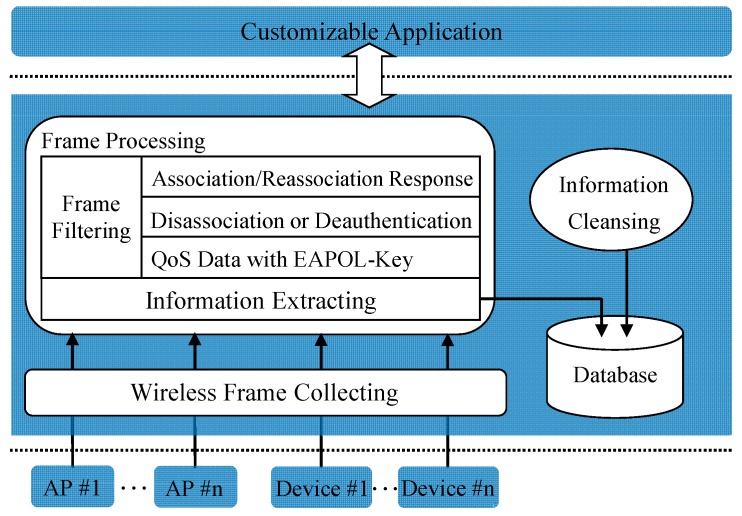
Overview of system architecture.

The aim of this section is to describe in details the system. The proposed system is shown in Figure 4 and is explained below. The system has three key components, namely, *wireless frame collecting*, *frame processing*, and *information storage*. Wireless frame collecting captures 802.11 MAC frames in Wi-Fi networks. Frame processing is responsible for selecting frames related to people’s access behaviors from the captured frames and extracting useful information from each selected frame. Information storage stores the extracted information into database. Furthermore, the two-sized sliding window algorithm and the judging policy for access validness are implemented in *information cleansing*.

### 3.1. Wireless Frame Collecting

Capturing 802.11 MAC frames in Wi-Fi networks can be realized via the monitor mode of the wireless network adapter. Monitor mode [36] allows a wireless network adapter to monitor all traffic received from Wi-Fi networks in a non-intrusive manner. Usually whether a wireless adapter is able to operate in monitor mode or not depends on its driver, firmware, and chipset features. Different operating systems show different features for supporting monitor mode. For example, the network driver interface specification on older Windows versions does not support any extensions for wireless monitor mode, whereas Windows Vista and later versions of Windows do. Some Unix-like operating systems provide interfaces for many drivers (i.e., 802.11 drivers) that support monitor mode, such as Linux, FreeBSD, NetBSD, OpenBSD, DragonFly Bsd, and Mac OS X 10.4 and later releases.

The way to enable the monitor mode varies from the type of the operating system. For example, the monitor mode of a wireless network adapter can be enabled by command lines on Linux, or by invoking function libraries that comprises application programming interface (API) for capturing network traffic (i.e., Winpcap on Windows, Libpcap on Linux). Besides, some packet analyzer applications such as OmniPeek, CommView, and Wireshark (i.e., on Windows or Linux) can also be utilized.

After enabling the monitor mode, a computer with a wireless network adapter can monitor all wireless frames that flow through the adapter in Wi-Fi networks. The APIs provided by function libraries, or packet analyzers can be utilized to store the captured packets in pcap files. In this article, Linux is used as the operating system, under which the wireless network adapter with monitor mode works. And APIs of function libraries are invoked to operate the adapter and process the collected frames.

### 3.2. Frame Processing

**Figure 5:**
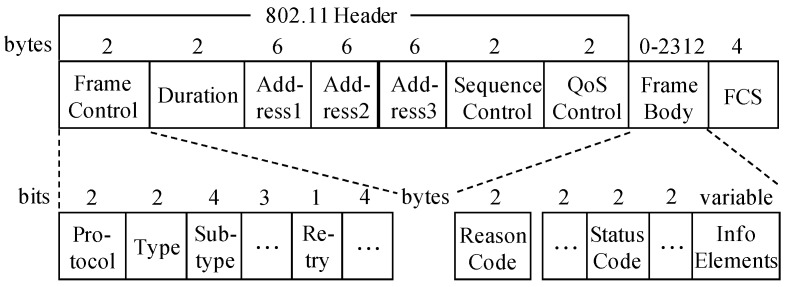
Generic 802.11 frame format.

Frame processing includes *frame filtering* and *information extracting*. In frame filtering, we select frames related to people’s access behaviors from captured frames, including Association/Re-association Response frames, Disassociation frames, De-authentication frames, and QoS Data frames that encapsulate EAPOL-Key messages, based on the frame format. These frames use generic frame format [37], and each can be identified by the six-bit Type and Subtype fields, as shown in Figure 5. Table 2 shows how the Type and Subtype identifiers are used for different frames. In Table 2, bit strings are written most-significant bit first, which is the reverse of the order used in Figure 5.

**Figure 6:**
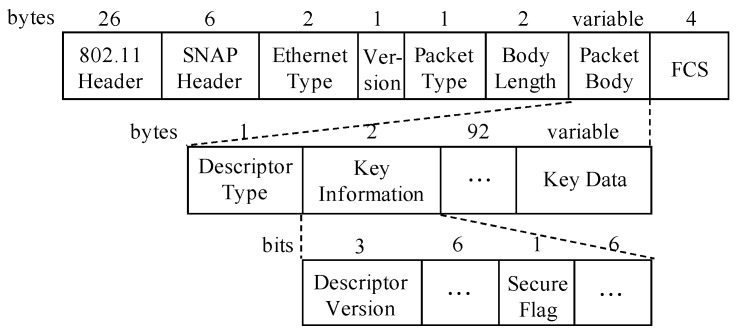
Format of the QoS Data frame with the EAPOL-Key message.

To identify which EAPOL-Key message a QoS Data frame encapsulates, the Ethernet Type field and the Secure Flag field in the QoS Data frame can be used, as shown in Figure 6. The code 0x888e is assigned to EAPOL, and the Secure Flag is set to bit 1 when the message is EAPOL-Key 3 or EAPOL-Key 4. Therefore, we can distinguish different QoS Data frames using above description.

Then in information extracting, useful information from each selected frame is extracted. As shown in Table 2, each frame includes three address fields, that is, destination address (DA), source address (SA), and basic service set identifier (BSSID). BSSID represents the MAC address of an AP. By comparing BSSID with the DA or SA, the MAC addresses and the transmission directions of the Wi-Fi device and the AP can be determined. Furthermore, the occurrence time of the access and exit event on the Wi-Fi device side can be obtained according to the assumptions defined in Section 2. From time to time, frames may be retransmitted. The Retry field of the retransmitted frame is set to bit 1. Besides, the two-byte Status Code field of the Association/Re-association Response frame is set to 0 when an association operation succeeds and nonzero on failure. While the two-byte Reason Code field of the Disassociation or De-authentication frame indicates the reason of an exit operation. Status codes and reason codes have been standardized, which can be referenced in [37].

### 3.3. Information Storage

After frame processing, the information is stored into database. Table 3 illustrates the information fields stored in database in this article. The *ValiFlag* field is used to denote the validness of an access operation. If an access operation is valid, then the field is set to 1, otherwise, set to 0. The *InOut* field is set to *eapol12* for the EAPOL-Key 1 or EAPOL-Key 2 message, and is set to *eapol34* for the EAPOL-Key 3 or EAPOL-Key 4 message.

The access/exit information of different Wi-Fi devices stored in database is in chronological order based on the time when the system receives each access/exit frame. The information is not completely reliable. The loss and retransmission of the 802.11 MAC frame, caused by inevitable noises and electromagnetic interferences result in some unreliable information in database. Here we only consider the items stored in database with same *StaAdd* and *ApAdd*. There are five possible cases:

*Case* *1:* If the *InOut* fields of two adjacent items are set to *in*, then whatever the *RetrFlag* fields are set to, the first item is always unreliable. When the *RetrFlag* field of the second item is set to 0, it means the corresponding exit frame to the first item is lost. If the *RetrFlag* field the second item is set to 1, the first item is useless, and should be abandoned.
*Case* *2:* If the *InOut* fields of two adjacent items are different, we think they are both reliable.
*Case* *3:* If the *InOut* fields of two adjacent items are *out*, and the *RetrFlag* field of the second item is set to 0, then the second item is unreliable, because the corresponding access frame is lost.
*Case* *4:* For three adjacent items, if the *InOut* fields of the latter two are set to *out*, and the *RetrFlag* fields of the latter two are set to 0 and 1 respectively, then the unreliable item depends on the first item: (1) if the *InOut* field of the first item is set to *in*, then the second item is unreliable, because only the last retransmission is considered; and (2) if the *InOut* field of the first item is set to *out*, both of the second and third items are unreliable, and if there are more items after the third item, of which the *InOut* fields are set to *out* and the *RetrFlag* fields are set to 1, then these items are also unreliable, because the corresponding access frames are missing.
*Case* *5:* For three adjacent items, if the *InOut* fields of the latter two are set to *out*, and the *RetrFlag* fields are both set to 1, then no matter what the first item is, the second item is always unreliable. The concrete details are similar to Case 4, not tired in words here.

Aiming at the above cases, two-sized sliding window algorithm is proposed as the correcting mechanism. Each time the items with same *StaAdd* and *ApAdd* in database, denoted by set $S=\left\{s_{0},s_{1},\ldots \right\}$, are considered. Note that the items are still in chronological order. Then each two adjacent items in S from beginning to end are compared, and the unreliable items are removed. Let $L=\left\{l_{0},l_{1},\ldots \right\}$ be the unreliable item set. Algorithm 1 presents the pseudo-code of two-sized sliding window algorithm.
**Algorithm 1:** two-sized sliding window algorithm
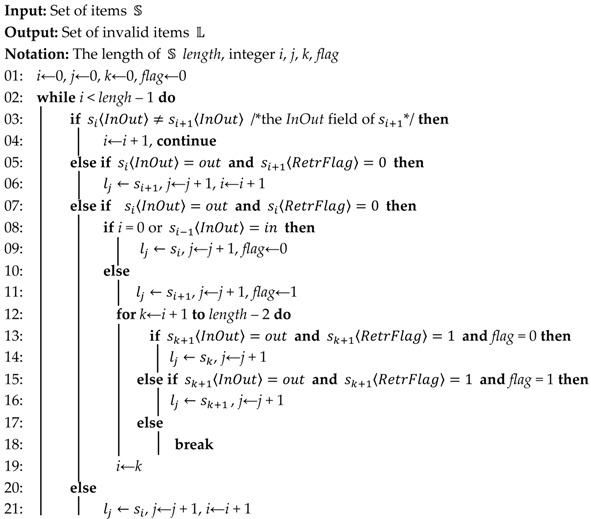


However, there may exist an exceptional case: after above algorithm, in set S, the *InOut* field of the first item is *out*, or the *InOut* field of the last item is *in*. These items are unreliable because we cannot be sure whether the corresponding access or exit frames are lost or not, they should be removed from database.
**Algorithm 2:** Judging policy for access validness
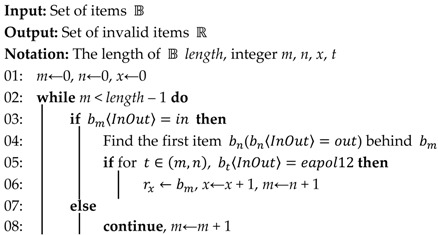


Algorithm 2 presents the pseudo-code of judging policy for access validness. The items with same *StaAdd* and *ApAdd* in database are stored into a set. Let $B=\left\{b_{0},b_{1},\ldots \right\}$ be the set. The output set $\mathbb{R}=\left\{r_{0},r_{1}\ldots \right\}$ comprises the invalid access items. If an access operation is valid, the *ValiFlag* field of the access item is set to 1; otherwise set it to 0.

## 4. Experimental Demonstration

This section mainly focuses on the implementation and evaluation of the system we designed. The major emphasis of the experiment is placed on the accuracy verification of the tracked information. It makes sense to confirm this issue for further studying the feasibility of the proposed system. To this end, the system was prototyped and experiments were conducted on single target AP within real outdoor Wi-Fi networks. 

### 4.1. Prototype Setup

**Figure 7:**
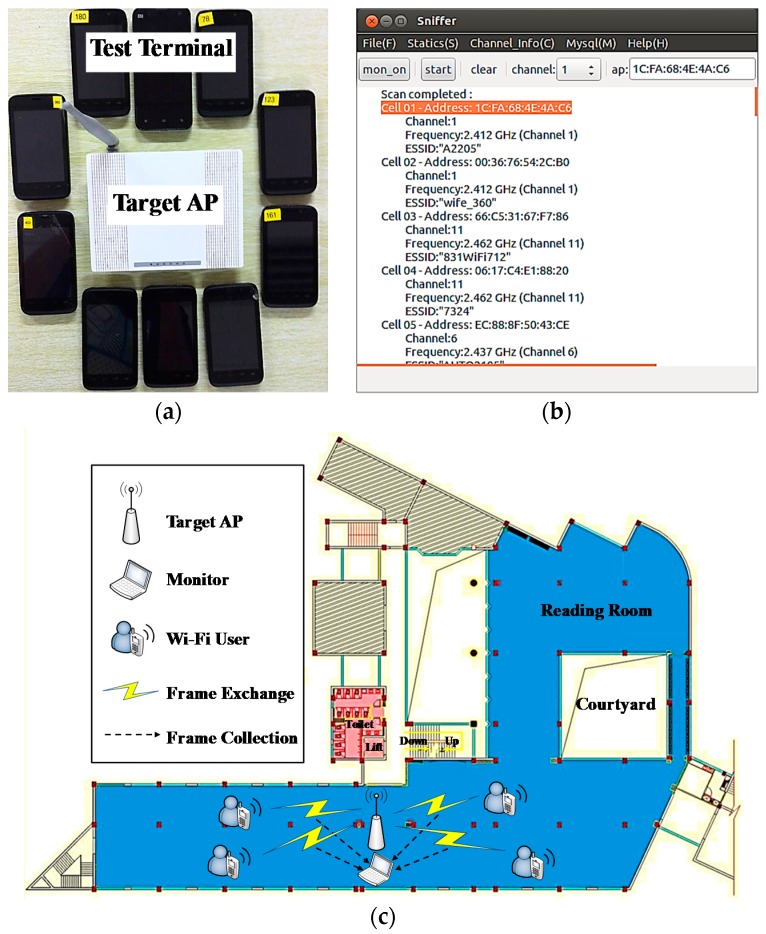
Experimental setup: (**a**) Experiment equipment; (**b**) The GUI of the monitor; (**c**) The floor plan of the experiment.

The proposed system is implemented on a HP Pivilion g4 PC equipped with a 2.66 GHz Intel Core 2 Duo Processor. The PC is equipped with the Qualcomm Atheros AR9285 802.11b/g/n Wi-Fi Adapter. The operating system (OS) is 32-bit Ubuntu LTS 12.04. Libpcap [38], a network packet capture library, is invoked to capture Wi-Fi messages. The captured information is stored into MySQL [39]. GTK + toolkit [40] is used to create graphical user interface (GUI), as shown in Figure 7b.

We have evaluated the system in Chongqing University Library (CUL), China, in which some APs had been deployed. Most these APs were encrypted by WPA/WPA2-PSK, and less were unencrypted. We chose encrypted AP as the experiment target. The reason why we did this choice instead of unencrypted AP is that the access procedures on the encrypted AP comprise those on the unencrypted AP as shown in Figure 3. In other words, the latter can be regarded as a special case of the former. Therefore, we can conduct experiments on encrypted AP for the accuracy verification of the tracked information without loss of generality. The target encrypted AP we chose operates on the frequency of 2.412 GHz. For the issue of monitor placement, we have placed the system near the target AP to increase chances of people being monitored. The floor plan of CUL is shown in Figure 7c. Note that the wireless environment is complicated and changeable, especially in doors. The Wi-Fi signal always suffer the influence of unpredictable path loss and multipath fading [40]. One way to compensate this limitation is to model the wireless environment. However, when the tracking environment changes, the model built before is useless, a new model has to be constructed. In our work, we neglect the environment factor.

We choose smartphones as the Wi-Fi devices. We prepared ten smartphone prototypes with 1.6 GHz Intel Atom Z2460 processors as Wi-Fi devices and distributed them to ten participants, as shown in Figure 7a. Note that the type of the Wi-Fi device can influence the tracking accuracy. For instance, the Wi-Fi devices equipped with iOS 8 randomize the MAC addresses while scanning for Wi-Fi networks [41]. That means the MAC address used for Wi-Fi scans may not always be the device’s real (universal) address. This may cause some interference for identifying people. All ten participants knew the password to access the target AP beforehand. They accessed the target AP randomly, and each of them had to access and exit from the target AP at least once respectively. Furthermore, the access and exit time information was recorded by the small android application running on the smartphone. The application runs as a background process to monitor the Wi-Fi connection status.

The time we did the experiment was from 7 p.m. to 8 p.m., during which the flow of people is relatively large so the wireless environment is crowded and complicated. This prerequisite ensures that our experiment was conducted in a common scenario.

### 4.2. Experimental Results

**Figure 8:**
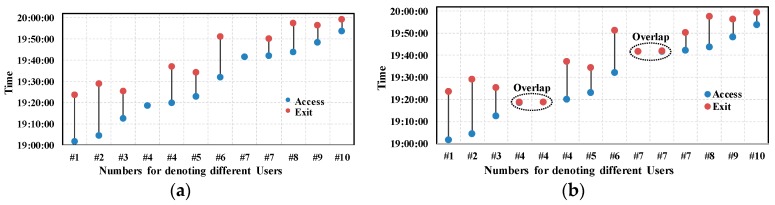
Experimental results: (**a**) Records of the experiment; (**b**) Output information of the system.

Figure 8 shows the comparison between the original records and the output information of the system. Figure 8a illustrates the original records during the experiment. The records are mainly about the access and exit time of each people, and the time is accurate to seconds. As we can see from Figure 8a, each user accessed the target AP only once, except for user 4 and user 7. In our experiment, we arranged user 4 and user 7 to access the AP twice. In addition, both of user 4 and user 7 used wrong passwords on purpose when the first time they accessed. Using wrong password can result in 4-way handshake failure in Figure 3. As we mentioned before, if 4-way handshake fails, the Wi-Fi device will continue to attempt to access the AP several times, rather than giving up. As shown in Figure 8b, after the first access operation failed, the device of user 4 or user 7 did one more attempt. However, the access still failed. After that, the device or the AP aborted the association. Under normal circumstances, each user accessed the AP at a certain instant, and exited at another instant. However, as illustrated in Figure 8b, the access and exit time of user 4 or user 7 are overlapped. This is because the access and exit operations occurred almost simultaneously when access failed.

As we can see from Figure 8, the output information of the proposed system shows a close agreement with the original experiment records, which fairly verifies the feasibility of the proposed system and correctness of the proposed two-size sliding window algorithm. Furthermore, by comparing the time information recorded on the smartphones and that recorded by the system, we observe that the system can correctly track people’s access information within a one-second delay. Such low delay can be neglected in the researches for characterizing people’s access behaviors, in which the minimum unit of the time is one minute or one hour. 

**Figure 9:**
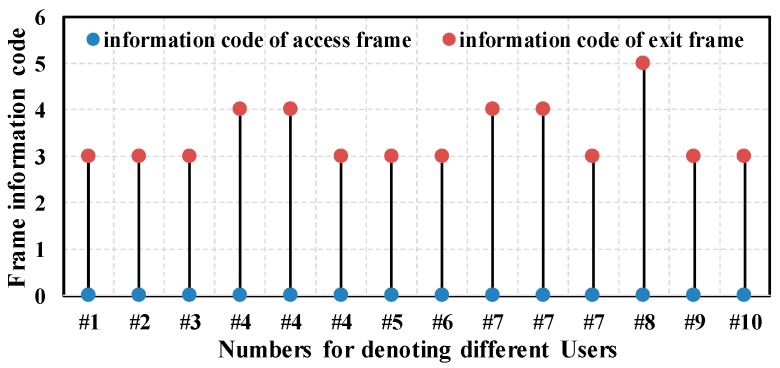
Frame type and transmission direction.

In addition to above information, there are some other details about access and exit operations of the Wi-Fi devices. Figure 9 illustrates the type and transmission direction of access or exit frames for each Wi-Fi device. For the sake of simplicity, we encode the information into different codes, as shown in Table 4. Note that all access frames are Association Response frames sent by the target AP, not including Re-association Response frames. This indicates there was no roam or hand-off happens.

Most exit information codes are 3. We can infer that the Disassociation frame is commonly used to abort a connection. Furthermore, the Wi-Fi device of user 8 sent the De-authentication frame to abort the connection. We observe that the first two exit operations of the Wi-Fi devices of user 4 and user 7 used the De-authentication frame to abort the connection. More importantly, the frames were sent by the target AP, not by the two Wi-Fi devices. We can infer that because the 4-way handshakes failed, the target AP could not distribute keys to the two Wi-Fi devices, and it had to end the connections.

**Figure 10:**
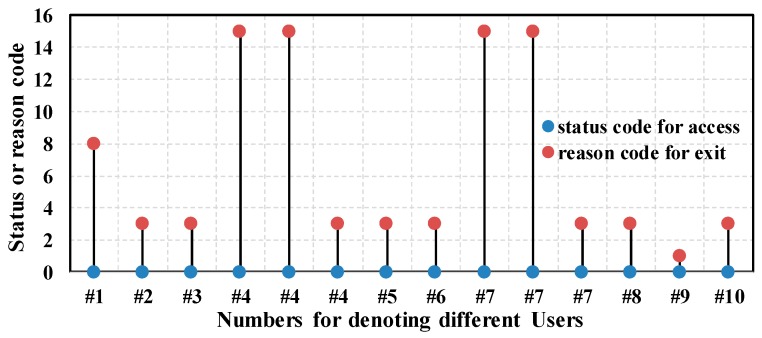
Status codes and reason codes.

As shown in Figure 10, the status codes of the access frames are 0, which means all the association operations succeeded. But there are different reasons for the exit operation of each Wi-Fi device. According to the 802.11 standard [37], code 8 indicates disassociation because sending Wi-Fi device is leaving or has left from the basic service set (BSS) in which the target AP is located. Code 3 indicates de-authentication because sending Wi-Fi device is leaving or has left from the extended service set (ESS). Because we only used one AP, the BSS and ESS are identical. We can infer these users might disable the Wi-Fi or they left the coverage of the AP. Note that for the first two exit operations of user 4 and user 7, the reason code is 15 indicates the 4-way handshakes are timeout, which matches what we expected before. Note that for user 9, the reason code is 1, which indicates the reason is unspecified.

**Figure 11:**
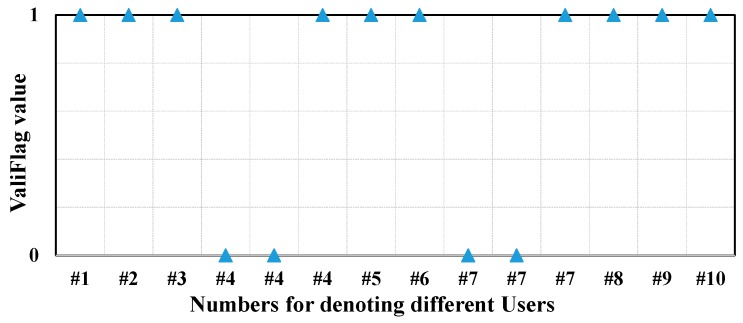
*ValiFlag* value for the access operation of each device.

To examine the accuracy of Algorithm 2, we also observe the *ValiFlag* field. As we mentioned before, the *ValiFlag* field in database is used to denote the validness of an access operation. If an access operation is valid, the field is set to 1, otherwise, set to 0. Figure 11 gives an intuitive illustration of the value of *ValiFlag* after the proposed algorithms. As shown in Figure 11, the first two access operations of user 4 and user 7 are invalid, which is in well agreement with the analysis before.

## 5. Conclusions and Outlook

### 5.1. Conclusions

In this article, we studied a method of tracking people’s behaviors. First, we summarized the shortcomings of traditional methods, and proposed a Wi-Fi-based passive CPSS method. Then, to validate the feasibility of the proposed method, a third-party system was designed targeting for tracking people’s access behaviors in Wi-Fi networks. The system operates in a non-intrusive, flexible, and simplified manner. We also described the details of the system implementation, including a two-sized sliding window algorithm used to eliminate the interference information caused by the loss and retransmission of the IEEE 802.11 MAC frame, and a policy to judge the validity of an access operation. Finally, the system was implemented on an off-the-shelf PC, and evaluated in real Wi-Fi networks. Real-world experiments showed the feasibility of the proposed method. The proposed system could correctly track people’s access behaviors within a one-second delay.

The collected traces can be utilized to characterize people’s individual behavior or group behaviors [28,29], which is beneficial for modelling, managing, leveraging and designing efficient mobile networks. By conducting aggregated statistics and modeling analysis on access and exit information, people’s presence activities, mobility, and association preferences in the area of interest can be evaluated [28]. Combing the status code and reason code information, more in-depth insights into association behaviors can be provided, which contributes to the statistics and models. In addition, by the aid of frame type and transmission direction information, hand-off events can be identified and studied. In a word, through this system we ease the way to collect detailed access traces with low cost and high flexibility, such that brings us closer to understanding people’s access patterns and behaviors in Wi-Fi networks. 

### 5.2. Outlook

For future work, we will consider large-scale experiments in the environment with multiple APs such as university campus or office buildings. There are some open issues which need to be addressed:

- The effective access behavior tracking will require deployment of several wireless monitoring systems. In this case, the density and topology of deployment can influence the accuracy [42]. Obviously, the more monitors are deployed, the higher accuracy can be realized, but as the amount of monitors increases, the cost goes up. Therefore, how to design the optimized topology of the monitor to minimize the cost is a big challenge. It is possible to tackle this challenge in future by using the knowledge of geometry.
- In addition, the traces captured by multiple monitors will require additional data sanitization techniques to address the scenario when a transmission from a single Wi-Fi device is observed on multiple monitors. cloud-based processing architecture will be explored, in which cloud servers are utilized to perform centralized processing for the traces captured by multiple monitors.
- Furthermore, a single system may be utilized to monitor multiple APs with different operating frequencies. In this case, some mechanisms like dynamic frequency switching must be considered, in which a system switches to different frequencies in different time slot to perform tracking. In this case, how to design the switching policy to maximize the tracking accuracy can be a big challenge.
- In some scenarios, there are massive people trying to access single AP. The network becomes very congested. Therefore, the probability of missing tracking gets higher. Some solutions need to be proposed, such as using multiple monitors and designing reasonable deployment structures to mitigate the burden.

Toward this end, more tests need to be conducted to further validate the performance of the proposed system. Moreover, more practical potential applications for the proposed system will be experimentally demonstrated, such as passenger flow statistics, precision marketing, and criminal hunting.

- Passenger flow statistics. By counting the number of different MAC addresses extracted from the receiving Wi-Fi frames, the proposed system can be utilized to conduct statistics on passenger flow in some operating regions, such as tourist attractions. Comparing to traditional statistical methods, such as artificial statistics and camera statistics, the proposed system can save the labor cost and lower the statistics difficulty. However, there are some open issues exist. For example, how to guarantee the Wi-Fi enabling of all the Wi-Fi devices to avoid missing of statistics. In addition, some people carry more than one Wi-Fi device, how to avoid duplication of statistics on these people.
- Precision marketing. By sensing the Wi-Fi signals from the Wi-Fi devices carried by people, people’s presence can be recognized. Furthermore, by deploying a set of the proposed systems in the area of interest, people’s movement trajectories can be tracked. This would be very helpful for precision marketing in retail stores or shopping malls. For example, changing the store layout according to the people’s moving trajectories to conducting marketing campaigns and advertising deliveries in the popular paths. In addition, by the analysis on the MAC addresses and time information the system tracked, the proportion of new and old customers, visiting cycle, and customer activity are easy to obtained, which can be utilized for grasping the composition of customers to provide references for the marketing strategy adjustment. In this application, how to consider the density and topology of the multiple systems to minimize deployment cost and maximize tracking accuracy is a big challenge.
- Criminal hunting. Our system can also be used as a criminal hunting technology. In large scale deployment, the tracked information such as MAC addresses of criminals’ Wi-Fi devices on each sensing node is delivered to the central servers combing the time information and location tags of each node. By comparing the tracked information with that in the database built beforehand, the location or the trajectory of the criminal can be tracked. Compared to the hunting technology based on base station positioning, using the proposed system can enormously improve the positioning precision. Usually, people possess many Wi-Fi devices with multiple different MAC addresses, while only one cellphone number. Therefore, compared to the hunting technology based on base station positioning, using the proposed system can also increase the success probability for criminal hunting. There also exists a challenge. For the Wi-Fi devices equipped with iOS 8, the MAC addresses are randomized while scanning for Wi-Fi networks. That means the MAC address used for Wi-Fi scans may not always be the device’s real address, which causes some interference for criminal hunting.

## Figures and Tables

**Table 1:** Comparison between different people tracking technologies.

Tracking Methods	Pros	Cons
GPS [17] (participatory)	High positioning precision Low-cost to deploy	Need people to participate Rarely works indoors
Wi-Fi [21] (participatory)	High positioning precision Low-cost to deploy	Need people to participate Need special software
Camera [13,14] (passive)	Non-intrusive High positioning precision No devices carried by people	LOS Inflexible deployment
RFID [15] (passive)	NLOS Flexible deployment High positioning precision	Intrusive Dedicated devices carried by people
ZigBee [16] (passive)	NLOS Flexible deployment High positioning precision	Intrusive Dedicated devices carried by people
Bluetooth [18] (passive)	NLOS Flexible deployment High positioning precision	Intrusive Dedicated devices carried by people
Cellular signal [19,20] (passive)	NLOS Wide Coverage Widely deployed infrastructures	Intrusive Poor positioning precision Inflexible deployment Common devices carried by people
Proposed system (passive)	Non-intrusive NLOS Flexible deployment Relatively high positioning precision	Common devices carried by people

**Table 2:** Information fields description.

Type	Subtype	Frame Name	Address1	Address2	Address3
00	0001	Association Response	Destination	Source	BSSID
00	0011	Reassociation Response	Destination	Source	BSSID
00	1010	Disassociation	Destination	Source	BSSID
00	1100	Deauthentication	Destination	Source	BSSID
10	1000	QoS Data(from AP)	Destination	BSSID	Source
10	1000	QoS Data(to AP)	BSSID	Source	Destination

**Table 3:** Information fields description.

Field Name	Description	Illustration
*StaAdd*	Mac address of station	0c:37:dc:d3:25:22
*ApAdd*	Mac address of AP	1c:fa:68:4e:4c:c6
*FrameInfo*	Frame type and direction	disassociation_from_ap
*RecTime*	Receiving time	2014-08-26 20:33:00
*InOut*	Access or exit	*in*/*out*/*eapol12*/*eapol34*
*StaCode*	Status code	0-65535/65536(default)
*ReaCode*	Reason code	0-65535/65536(default)
*RetrFlag*	Retransmitted or not	1(retransmit)/0(not)
*ValiFlag*	Access succeeds or not	1(succeed)/0(not)/65536(default)

**Table 4:** Frame Information Encoding.

Frame Information	Code
Association Response frame from AP to Wi-Fi Device	0
Re-association Response frame from AP to Wi-Fi Device	1
Disassociation frame from AP to Wi-Fi Device	2
Disassociation frame from Wi-Fi Device to AP	3
De-authentication frame from AP to Wi-Fi Device	4
De-authentication frame from Wi-Fi Device to AP	5
